# Integrating ZooMS and zooarchaeology: New data from the Uluzzian levels of Uluzzo C Rock Shelter, Roccia San Sebastiano cave and Riparo del Broion

**DOI:** 10.1371/journal.pone.0275614

**Published:** 2022-10-13

**Authors:** Sara Silvestrini, Federico Lugli, Matteo Romandini, Cristina Real, Eduardo Sommella, Emanuela Salviati, Simona Arrighi, Eugenio Bortolini, Carla Figus, Owen Alexander Higgins, Giulia Marciani, Gregorio Oxilia, Davide Delpiano, Antonino Vazzana, Marcello Piperno, Carlo Crescenzi, Pietro Campiglia, Carmine Collina, Marco Peresani, Enza Elena Spinapolice, Stefano Benazzi

**Affiliations:** 1 Dipartimento di Beni Culturali, Università di Bologna, Ravenna, Italy; 2 Departament de Prehistòria, Arqueologia i Història Antiga, Universitat de València, València, Spain; 3 Dipartimento di Farmacia, Università di Salerno, Fisciano, Salerno, Italy; 4 Dipartimento di Scienze Fisiche, della Terra e dell’Ambiente, Unità di Ricerca di Preistoria e Antropologia, Università di Siena, Siena, Italy; 5 Dipartimento di Studi Umanistici, Sezione di Scienze Preistoriche e Antropologiche, Università di Ferrara, Ferrara, Italy; 6 Museo Civico Archeologico Biagio Greco, Mondragone, Caserta, Italy; 7 Istituto di Geologia Ambientale e Geoingegneria, Consiglio Nazionale delle Ricerche, Milano, Italy; 8 Dipartimento di Scienze dell’Antichità, Università degli Studi di Roma “La Sapienza”, Roma, Italy; Griffith University, AUSTRALIA

## Abstract

In this study we explore the potential of combining traditional zooarchaeological determination and proteomic identification of morphologically non-diagnostic bone fragments (ZooMS) collected from the Uluzzian levels of three Italian sites: Uluzzo C Rock Shelter, Roccia San Sebastiano cave, and Riparo del Broion. Moreover, we obtained glutamine deamidation ratios for all the contexts analysed during routine ZooMS screening of faunal samples, giving information on collagen preservation. We designed a selection protocol that maximizes the efficiency of the proteomics analyses by excluding particularly compromised fragments (e.g. from taphonomic processes), and that aims to identify new human fragments by favouring bones showing morphological traits more similar to *Homo*. ZooMS consistently provided taxonomic information in agreement with the faunal spectra outlined by traditional zooarchaeology. Our approach allows us to delineate and appreciate differences between the analysed contexts, particularly between the northern and southern sites, related to faunal, environmental, and climate composition, although no human remains were identified. We reconstructed the faunal assemblage of the different sites, giving voice to morphologically undiagnostic bone fragments. Thus, the combination of these analyses provides a more complete picture of the faunal assemblage and of the paleoenvironment during the Middle-Upper Palaeolithic transition in Italy.

## Introduction

High fragmentation of bone assemblages is a common characteristic for Palaeolithic contexts. Such a pattern is caused by different agents: depositional and taphonomic processes [1,2], butchering and exploitation practices by humans [3] and carnivore activity [4]. These fragmented remains are mainly taxonomically indeterminate and, although they are often evidence of the action of different agents (humans, carnivores), they do not provide specific information about hunting activities (e.g. type of prey, transport decision, anatomical selection) or paleoclimatic and paleoenvironmental conditions. Peptide mass fingerprinting of these bone fragments allows us to overcome these issues [5]. ZooMS (Zooarchaeology by Mass Spectrometry) was developed as a method to discriminate tissue rich in collagen type I from a taxonomic point of view [5]. This application is based on the fact that the amino acid sequence of collagen type I varies across different taxonomic groups. The ZooMS approach is increasingly used to identify at a taxonomical level highly fragmented and/or morphologically unidentifiable faunal remains, and to complete the taxonomic spectrum [6–8]. Moreover, it can be applied to Palaeolithic contexts to screen for the presence of human remains [9,10]. Specifically, the unavailability of taxonomic information is significant in Middle-Upper Palaeolithic contexts, which play a crucial role in understanding the development and diffusion of modern humans and disclosing the timing and causes of the extinction of Neanderthals [11–13]. Mediterranean Europe, and Italy in particular, is a key area for the comprehension of this period, due to the presence of relevant archaeological sites dated to the Middle-Upper Palaeolithic transition and the occurrence of several technocomplexes, i.e., Mousterian, Uluzzian, and Protoaurignacian [14]. From a technological point of view, the Uluzzian is characterised by its own originality and consistency [15,16] both expressed by the production of specific classes of bone tools and ornaments [17–22] as well as by a specific conceptualisation of the stone tool production. In fact, the Uluzzian lithic production is generally characterised by: the use of mostly local raw materials; additional concepts of debitage (unidirectional reduction sequence); the absence of integrated concepts of debitage, (i.e. Levallois); a massive use of the bipolar technique on anvil; the production of flakes and bladelets with several morphologies; a low degree of standardisation of the products; the presence of lunates and end-scrapers among the retouched tools [14,15,23,24]. Zooarchaeological data for the Middle to Upper Palaeolithic transition suggest that human adaptation strategies changed over time to manage changes in the ecological conditions and to face uncertainties in the availability of resources [25]. In southern Italy, has been recorded a marked change in the exploitation of ungulates between late Mousterian and the beginning of the Upper Palaeolithic [25,26], whereas in northern Italy a greater variety of animal processing techniques was already present since the late Mousterian [25].

### The pivotal role of the Uluzzian and research questions

The identification of hominin bones dated to the Middle-Upper Palaeolithic transition is essential to disentangle the dynamic related to the demise of Neanderthals and the modern human peopling of Europe and specifically Italy. Currently, in Italy, only a small number of late Neanderthals and early modern human specimens is available e.g., [27–29]. Only one Italian site has yielded human remains found in association to the Uluzzian materials, it is the case of Grotta del Cavallo [18,28] see contra [30]. For all these reasons, our understanding of the Uluzzian is far from being complete and the in-depth investigation of bone fragments from Uluzzian sites is a task must be tackled to better define the association between this techno-complex and a specific human species, as well as to better understand the hunting strategies and interactions between humans and animals.

Here, we explore the potential of combining traditional zooarchaeological determination and proteomic identification of morphologically non-diagnostic bone fragments collected from the Uluzzian levels of three Italian sites: Uluzzo C Rock Shelter, Roccia San Sebastiano cave and Riparo del Broion. To the best of our knowledge, this is the first time Uluzzian levels from three different contexts have been tested by integrating ZooMS and morphological analyses. We also obtained glutamine deamidation ratios for all the contexts analysed during routine ZooMS screening of faunal samples. Deamidation has been proposed to: i) be a relative dating method to establish the chronological relationship between samples derived from different archaeological contexts or different stratigraphic units [31–34], although [35] suggested that glutamine deamidation is too susceptible to environmental conditions to be a reliable marker of chronological age; ii) determine the homogeneity of a set of samples iii) define the rate of collagen degradation or preservation [7,31,36]. In this study, this last point was considered. Furthermore, we designed and propose a sample-selection protocol with two main goals: (i) maximize the efficiency of the proteomics analyses by choosing specimens responding to specific morphological characteristics (see Methods) and avoiding ones with a compromised taphonomic state (e.g. concretions, manganese coating) and (ii) identify new human fragments in the osteological assemblage. Our approach highlights differences in the faunal and environmental composition between the contexts under analysis.

## Materials and methods

### Archaeological contexts

**Fig 1:**
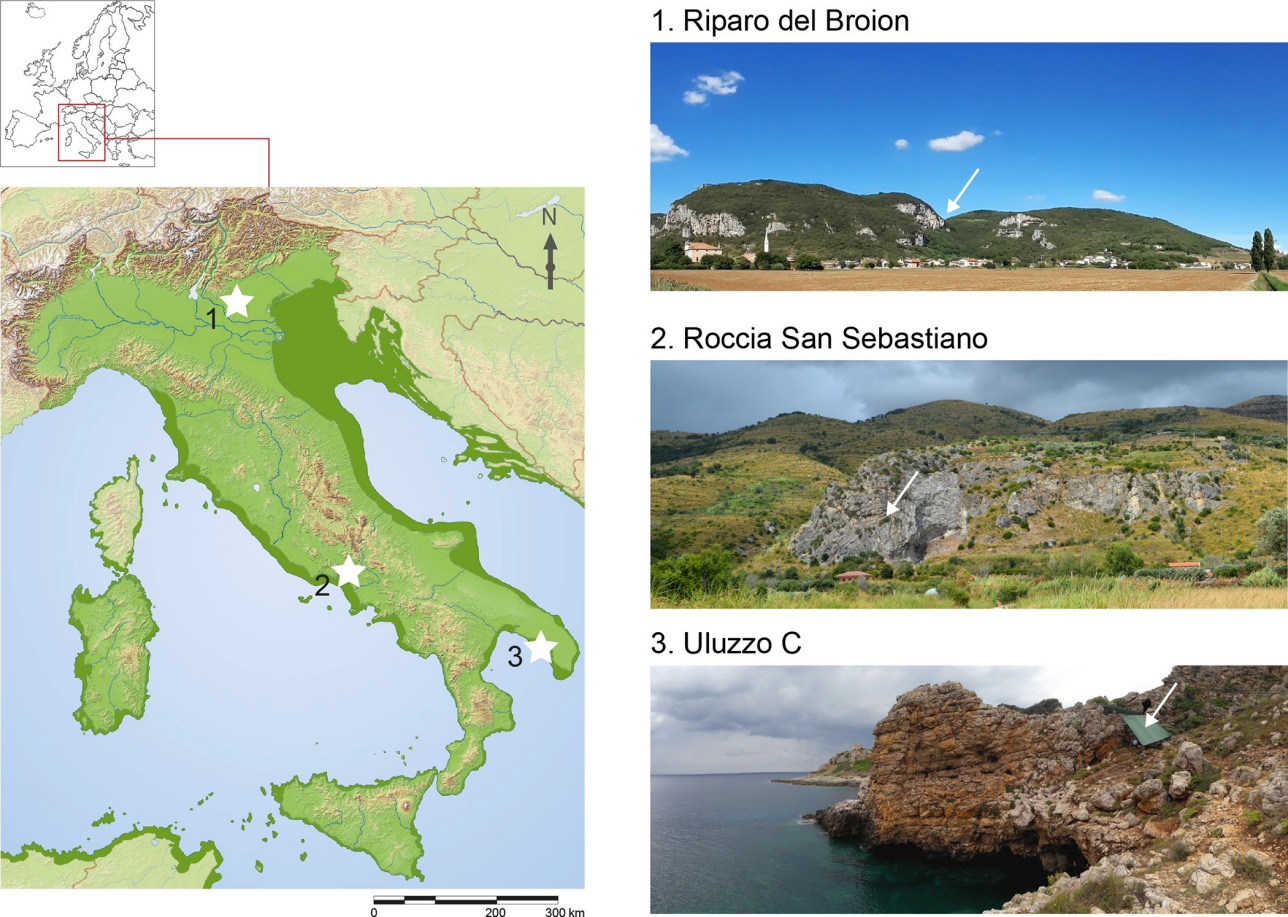
Localization of the Uluzzian sites analysed. 1. Riparo del Broion (Veneto, North Italy) 2. Roccia San Sebastiano cave (Campania, South Italy) 3. Uluzzo C Rock Shelter (Apulia, South Italy). Map of Italy during MIS 3, modified from [15].

The sites included in this work are located in three different areas of Italy: Uluzzo C Rock Shelter in the south-eastern (Ionian area), Roccia San Sebastiano cave in the south-western (Tyrrhenian Area), and Riparo del Broion in the north (Fig 1).

Uluzzo C Rock Shelter (Fig 1) is located in the Parco Naturale di Porto Selvaggio (Nardò, Lecce, southern Italy), on the western side of the Apulian Coast facing the Ionian Sea (40° 9’27.84″N 17°57’35.34″E). From a cultural point of view, the stratigraphic sequence of Uluzzo C includes a Romanellian layer (A); a sterile layer (B); two Uluzzian layers (C, D); a layer composed by a mixture of Upper Palaeolithic and Mousterian deposits (E); and a large sequence of Mousterian layers (F-L) [37–39]. This sequence was confirmed by the recent excavations, as well as by sedimentological and micromorphological analyses (for a detailed description see [40]).

The latest investigations (2016–2019) involved the stratigraphic excavation of SUs 3, 15+17 (published in [24]) and SUs 21+22+23+25 which were identified as three distinct phases of the same level of occupation characterised by Uluzzian lithic industry [24]. Based on the single-grain OSL chronology, the grand weighted mean age for the Uluzzian occupations—layers C, D and E (OSL samples ULOC 3, 4, and 5)—is 40.6±1.4 ka [40].

Roccia San Sebastiano cave (Fig 1) is located in the Municipality of Mondragone (Caserta, north-western Campania). It is a tectonic-karstic cave that opens at the foot of the southern slope of Mount Massico. The excavated archaeological sequence (visible in the reference trench E14-15) can be divided into three main units (labelled Units 1 to 3) based on their overall stratigraphic features and on the archaeological materials: Unit 1 includes Recent Gravettian, Gravettian and Early Gravettian layers whereas Unit 2 comprises Initial Gravettian and Aurignacian layers. Unit 3 is composed of two sub-units, an Uluzzian (which corresponds to t27-t28 in the E14-E15 trench; t18-t20 in F14 trench and t16-t18 in E16 trench) and Mousterian. The oldest date for the stratigraphic sequence in E14-E15 (R_Date Rome-2111) is 43,680–42,190 (68.2%) cal BP– 44,740–41,700 (95.4%) cal BP based on a bone fragment collected in sub-unit Cg-t29-34 (Final Mousterian) [23]. The new chronological range obtained for the Uluzzian layer (trench F14 pits t19; ETH-99090.1.1) is 42,640–42,380 cal BP [41].

Riparo del Broion (Fig 1) is located in the municipality of Vicenza, on a karst plateau at 135 m asl on the Berici Mounts, along a rocky wall that connects the top of Monte Brosimo (327 m asl) to the plain. The shelter formed from thermoclastic processes and chemical dissolution [42]. During the archaeological excavations, a Pleistocene sequence with faunal remains and a cultural sequence characteristic of the Middle-Upper Palaeolithic transition were discovered [19,27,43,44]. To date, at least 20 stratigraphic units have been identified. Unit 1 was divided into seven subunits (levels), from 1a to 1g, and it displays the following sequence of lithic assemblages: Early Epigravettian (layers 1a-1b), Gravettian (1c-1d) and Uluzzian (1e-1f-1g), preceded by Mousterian (units 4+7, 9 and 11). Units 2 and 3 at the current state of research did not show any traces of anthropic activity. Level 1g was radiocarbon dated to 44,421 (95.4%) 41,872 cal BP [19].

### Zooarchaeological analysis

Taxonomic and skeletal identification was based on the reference collection stored at the Laboratory of Osteoarchaeology and Palaeoanthropology (Bones Lab) of the Department of Cultural Heritage of the University of Bologna (Ravenna, Italy). All necessary permits were obtained for the described study, which complied with all relevant regulations. All bone fragments were counted and grouped by size (0–1 cm, 1–2 cm, 2–3 cm, 3–4 cm, 4–5 cm, >5 cm). All bone specimens were also grouped by body size class (large, medium-large, medium, medium-small, and small) based on cortical bone thickness and fragment size [45]. Faunal remains were classified as determinate (taxonomical and anatomical identification), indeterminate (solely anatomical identification), and unidentified. Unidentified bones are the ones for which neither the species nor the anatomical element were determined. To evaluate species abundance, we considered the number of identified specimens (NISP) [46] and the minimum number of individuals (MNI) [47]. We morphologically examined 20,392 bone fragments distributed as follows among the Uluzzian layers of: Uluzzo C Rock Shelter (n = 6,280), Roccia San Sebastiano cave (n = 1,998) and Riparo del Broion (n = 12,114). For Uluzzo C, we analysed all the remains coming from SUs 3, 15, 17, 21, 22, 23 and 25, which are divided among three distinct phases of the same occupation level. For Roccia San Sebastiano cave we examined all the specimens coming from the squares E16 (pits 16 and 17) and F14 (pits 18, 19, and 20). Whereas, for Riparo del Broion, we analysed all the bone fragments coming from layers 1g, 1f, and 1e (squares A, AA, AB, AC, AD, B, C and D). All the bone fragments are stored in Ravenna at the Bones Lab at the Department of Cultural Heritage of the University of Bologna.

### ZooMS analysis

Among the unidentified bone fragments, a sub-sample was selected to be analysed by ZooMS. The selection was performed according to the following criteria: (i) sample size larger than 2 cm; (ii) presence and arrangement of trabeculae within the bone fragment; (iii) scarce presence of taphonomic elements such as combustion, manganese, or concretion on the bone surface; (iv) position of the fragments as they were found during the excavation; (v) thickness, density, and porosity of the cortical bone. These criteria were designed considering the aim of this study, namely, to identify possible hominin remains and to maximize the yield of proteomic analyses by excluding fragments that were particularly compromised by different taphonomic processes. Finally, we selected and processed for ZooMS analysis: Uluzzo C Rock Shelter (n = 46), Roccia San Sebastiano (n = 55), and Riparo del Broion (n = 94). On these samples, glutamine deamidation ratios were also calculated. Selected specimens were sampled using a dentist drill (~10–30 mg of bone). For Uluzzo C and Riparo del Broion, we applied the protocol proposed by [5], whereas for Roccia San Sebastiano we tested the protocol designed by [48], using a warm (65°C) ammonium bicarbonate buffer (50 mM) to leach acid-insoluble bone collagen without prior demineralization. In this latter protocol, samples were first incubated in 500 μl of Ambic for 1 h, then briefly centrifuged and the supernatant discarded. The extraction was repeated for 3 h, and this supernatant was collected for trypsin digestion. As a first step, we tried to process all the samples by following van Doorn’s protocol [48] to be least invasive and to avoid inducing deamidation [49–51], though only for Roccia San Sebastiano produced taxonomic results. Thus, we proceeded by adding an initial phase of demineralization for approximately 24 h with 500 μl of 0.6 M HCl for the samples from Riparo del Broion and Uluzzo C. Following centrifugation and removal of the supernatant, the acid-insoluble residue was then gelatinised by heating at 65°C in 500 μl of Ambic for 3 h. Then, trypsin digestion was carried out for 18 h at 37°C using 0.5 μl of 1 μg/μl sequencing grade trypsin (Sigma). Enzymatic digestion was ended using 5 μl of 5% formic acid (FA), then the tryptic digests were purified and concentrated using C18 SpinTips (Thermo Scientific) and in-house STAGE tips (Empore^TM^). Peptide elution was performed with 50 μl of 50% acetonitrile (ACN)/0.1% FA (v/v). Extraction blanks were included throughout all analysis stages to monitor the introduction of potential contamination. Samples were dried overnight under a class 100 laminar flow hood. After samples were solubilised in 10 μl of ACN/H_2_O (50:50) + 0.1% TFA and sonicated for 30 seconds. The matrix (α-cyano-4-hydroxycinnamic (CHCA; Sigma) 10 mg/ml) was solubilised in the same solvent. 1 μl of CHCA matrix were mixed directly on a target steel plate with 1 μl of sample and left to dry in the air. The samples were then analysed in duplicate with a RapifleX Tissuetyper MALDI-ToF (Bruker Daltonics, Bremen, Germany) over a mass-to-charge range of 700–3500 *m*/*z*. Spectra were manually inspected and averaged using mMass [52], after setting a signal-to-noise ratio equal to 3.5, a baseline correction and smoothing with the default parameters (baseline correction precision 100 and relative offset 0; smoothing method Savitzky-Golay algorithm, window size 0.2 *m*/*z* and 1.5 cycles; peak picking with deisotoping tool, relative intensity threshold 0.5% and picking height 80%) [53]. Taxonomic identification was performed manually, comparing identified collagen peptides with a database of peptide markers for all European, Pleistocene medium to large sized mammals (published by [9], integrated with further peptide markers identified by [54,55]). MALDI-TOF-MS raw spectra can be found at Zenodo, doi: 10.5281/zenodo.6942800.

### Glutamine deamidation calculation

Deamidation ratios were calculated for all the analysed samples. Deamidation of glutamine and asparagine residues is a common, non-enzymatic modification that results in a +0.98402 mass shift caused by the conversion of a side-chain amide group with a carboxylate via a cyclic intermediate (glutarimide/succinimide) under alkaline and neutral conditions or direct hydrolysis under acidic conditions (or in the presence of metal cationic species) [35]. Since specific peptides are more useful than others for comparing deamidation rates between samples than others, peptides with m/z 1105.6 and 1706.7 were selected to obtain the percentage of deamidation [35]. The deamidation ratios were obtained using the R 4.0.1 *q2e* package [34,56], which includes the deconvolution of the isotopic pattern.

## Results

### Zooarchaeological analysis

#### Uluzzo C Rock Shelter

612 (9.7%) of 6,280 bones were taxonomically and anatomically identified (Table 1). The indeterminate group includes remains that were divided only by size. Among the determinate remains, ungulates are the most represented taxa, and six species were registered. *Cervus elaphus* (6.8%) is the main taxa within the ungulates. Other species such as *Sus scrofa* (3.1%) and *Equus ferus* (3.1%) are represented by a fair amount of bone elements, *Capreolus capreolus*, *Bos/Bison*, and *Rupicapra* sp. are represented by few remains (less than 2%). Carnivores include only 10.1% of the determinate remains but include several species of canids (*Canis lupus* and *Vulpes vulpes*, the latter being the main taxa found in this context) and felids (*Felis silvestris* and *Lynx lynx*). Different species of mustelids are also present: *Meles meles*, *Mustela nivalis*, and *Martes* sp. Among Lagomorpha, *Lepus* sp. is represented by eleven bones. The indeterminate remains are represented mainly by fragments of indeterminate animals (99.6%).

**Table 1:** Mammals NISP (Number of Identified Specimens), %NISP and MNI (Minimum Number of Individuals) for the analysed contexts. RB (Riparo del Broion), RSS (Roccia San Sebastiano cave) and UC (Uluzzo C Rock Shelter).

Site	RB			RSS			UC		
SU-Level	1e+1f+1g			F14 t.18, 19, 20 E16 t.16, 17, 18			3, 15+17, 21+22+23+25		
Taxa	NISP	%NISP	MNI	NISP	%NISP	MNI	NISP	%NISP	MNI
*Lepus* sp.	1	0.6	1	2	0.4	1	11	1.8	3
**Total Lagomorpha**	**1**	**0.6**	** **	**2**	**0.4**	** **	**11**	**1.8**	** **
*Canis lupus*	2	4.5	2	1	25	1	4	7.3	3
*Vulpes vulpes*	3	6.8	1	3	75		44	80	7
*Ursus arctos*	3	6.8	1						
*Ursus spelaeus*	9	20.5	2						
*Ursus* sp.	25	56.8	2						
*Mustela nivalis*							1	1.8	1
*Meles meles*	1	2.3	1				2	3.6	2
*Martes* sp.							1	1.8	1
*Felis silvestris*							2	3.6	2
*Felis* sp.	1	2.3	1						
*Lynx lynx*							1	1.8	1
Carnivora indet.									
**Total Carnivora**	**44**	**25.3**	** **	**4**	**0.8**	** **	**55**	**10.1**	** **
*Equus ferus*				15	2.9	1	17	3.1	5
*Sus scrofa*	11	8.5	4	14	2.8	3	17	3.1	4
*Megaloceros giganteus*	2	1.6	1	1	0.2	1			
*Alces alces*	2	1.6	1		0.2				
*Cervus elaphus*	7	5.4	2	112	22	5	37	6.8	8
*Dama dama*				2	0.4				
*Capreolus capreolus*	7	5.4	2	23	4.5		8	1.5	2
Cervidae	10	7.8	2	14	2.8	3	17	3.1	
*Bos primigenius*				6	1.2				
*Bos/Bison*	2	1.6	1	21	4.1	2	8	1.5	4
*Rupicapra rupicapra*				4	0.8	1			
*Rupicapra* sp.					0.0		3	0.5	2
*Capra ibex*	2	1.6	2		0.0				
Caprinae				3	0.6		6	1.1	
Ungulata	86	66.7		294	57.8		434	79.5	
**TOTAL Ungulata**	**129**	**74.1**		**509**	**98.8**		**546**	**89.2**	
**TOTAL NISP**	**174**	**1.4**	** **	**515**	**25.8**	** **	**612**	**9.7**	** **
Small sized mammals	11	0.1		42	2.8		37	0.7	
Small-medium sized mammals	2	0.0		40	2.7		39	0.7	
Medium sized mammals	6	0.1		179	12.1		45	0.8	
Medium-large sized mammals	32	0.3		510	34.4		51	0.9	
Medium/large sized mammals	25	0.2			0.0		8	0.1	
Large sized mammals	38	0.3		13	0.9		22	0.4	
Indet. Sized mammals	11826	99		699	47.1		5439	99.6	
**TOTAL INDET**	**11940**	**98.6**		**1483**	**74.2**		**5461**	**87.0**	
**TOTAL NR**	**12114**	** **	** **	**1998**	** **	** **	**6280**	** **	** **

#### Roccia San Sebastiano cave

515 (25.8%) of 1,998 bones were taxonomically and anatomically determined (Table 1). Ungulates (98.8%) clearly prevail over carnivores (0.8%) among the total of the determined remains. In general, the faunal record is mostly composed of medium/large-sized ungulates. The most represented taxon is *Cervus elaphus* (22%) (NISP = 112); relatively abundant are also the fragments of *Bos/Bison* (5.3%), *Capreolus capreolus* (4.5%), and *Sus scrofa* (2.8%). On the contrary, caprines, carnivores and small taxa are scarce. Very few remains of *Canis lupus* and *Lepus* sp. were registered. The indeterminate remains are represented mainly by bone fragments from medium-large-sized animals (34.4%) and fragments of indeterminate animals (47.1%).

#### Riparo del Broion

Only 174 (1.4%) of 12,114 bones were identified at both a taxonomic and anatomical levels (Table 1). In this site, carnivores are better represented (25.3%), mainly by *Ursus* sp. (56.8%) but also *Vulpes vulpes* (6.8%) and *Canis lupus* (4.5%) were registered. Ungulates, however, are still the main group (74.1%). Only one remain of *Lepus* sp., *Meles meles* and *Felis* sp. was identified. Among the ungulates, *Sus scrofa* (8.5%), *Cervus elaphus* (5.4%) and *Capreolus capreolus* (5.4%) are the most represented species. Considering other taxa, *Alces alces* (1.6%), *Megaloceros giganteus* (1.6%), *Bos/Bison* (1.6%) and *Capra ibex* (1.6%) are present but scarce. The indeterminate remains are almost entirely represented by fragments of indeterminate animals (99%).

### ZooMS analysis

#### Uluzzo C Rock Shelter

46 unidentified bone fragments were selected and analysed with ZooMS. Only 21 samples (46%) provided taxonomic information by comparing peptide marker series (A-G) with available vertebrate species [9,54,55]. Thus, 25 samples remain undetermined.

We taxonomically identified only 4 samples of *Equus ferus*, 2 of *Bos/Bison*, 1 of *Vulpes vulpes*, 1 of *Felis silvestris*, 4 of *Cervus elaphus*, 2 of Cervidae/*Equus ferus*, 2 of Bovidae/Caprinae/Cervidae, 3 of Caprinae/Cervidae and 2 ungulates. The latter were classified generically as ungulates due to the lack of distinctive diagnostic peptides (Table 2).

**Table 2:** ZooMS taxonomic determination for the three analysed contexts. RB (Riparo del Broion), RSS (Roccia San Sebastiano cave), UC (Uluzzo C Rock Shelter).

Site	RB	RSS	UC
SU-Level	1e+1f+1g	F14 t.18, 19, 20 E16 t.16, 17, 18	3, 15+17, 21+22+ 23+25
**Taxa**	** **	** **	** **
*Vulpes vulpes*			1
*Ursus* sp.	9		
*Felis silvestris*			1
*Ursus* sp./*Lynx lynx*	2		
*Canis lupus*		1	
Carnivora indet.	31		
**Total Carnivora**	**40**	** 1**	**2**
*Equus ferus*		6	4
*Sus scrofa*	5	4	
*Cervus elaphus*	1	2	4
Cervidae	9	6	
Cervidae/*Equus ferus*			2
*Bos/Bison*	1	9	2
Bovidae/Caprinae		3	
Bovidae/Caprinae/Cervidae		7	2
Caprinae/Cervidae	3		35
Ungulata	12	2	2
**TOTAL Ungulata**	**31**	**39**	**19**
*Sus scrofa*/Carnivora/Lepus sp.	2		
*Sus scrofa*/Carnivora	9	2	
**TOTAL INDETERMINATE**	**13**	**2**	** **
**TOTAL NISP**	**84**	**42**	**21**

#### Roccia San Sebastiano cave

55 unidentified bone fragments were selected and analysed by ZooMS, 42 samples (76%) provided taxonomic information and only 13 remain undetermined. The results allowed us to implement the morphological data from a quantitative point of view, but no new species was identified. Results are consistent with traditional zooarchaeological analysis: 1 *Canis lupus*, 6 *Equus ferus*, 4 *Sus scrofa*, 2 *Cervus elaphus*, 6 Cervidae, 9 *Bos/Bison*, 3 Bovidae/Caprinae, 7 Bovidae/Caprinae/Cervidae, 2 Carnivora*/Sus scrofa* and 2 ungulates were identified by ZooMS (Table 2). Based on the faunal composition of this site, the “Cervidae” category could comprise the following species: *Cervus elaphus*, *Capreolus capreolus*, *Dama dama*, *Megaloceros giganteus* and *Alces alces*. Small-sized species are not documented (e.g. *Lepus* sp., *Vulpes* sp., *Felis* sp.).

In cases of ambiguity in taxonomical identification (*Bos*/*Bison*/*Equus ferus*) through morphological analysis (e.g. RSS265), ZooMS analysis was useful to clarify the taxon of the sample. Conversely, 14 samples did not provide taxonomic information due to the lack of diagnostic peptides.

Two bone fragments with butchering traces were analysed by ZooMS: RSS176 (a fragment of coxal) and RSS94 (a fragment of calcaneus). The proteomic analysis was conclusive for RSS176, allowing to classify the fragment as *Sus scrofa*, which had already been morphologically attested in the context, as had the anthropogenic modifications on other *Sus* bones. As for RSS94, which had previously morphologically classified as a medium-sized carnivore, the MS spectrum showed the presence of ungulate-specific peptides (caprines and/or cervids). In this case, the morphological hypothesis was not corroborated, however, the taxonomic assignment by ZooMS is coherent with the data obtained from the site.

#### Riparo del Broion

84 of 94 tested samples (89%) retained useful information for taxonomic discrimination and only 10 remained unidentified. 9 samples could not be discriminated between cervids, caprines or bovids, 3 were classified as Caprinae/Cervidae, and 12 as generic ungulates. We placed an additional 9 samples under the generic classification of ‘Cervidae’. Based on the faunal composition, this category likely included: *Alces alces*, *Megaloceros giganteus*, *Cervus elaphus*, *Dama dama* and *Capreolus capreolus*. Further 31 bone fragments were diagnosed as generic ‘carnivora’ (Table 2). When the experimental spectra (n = 13) showed a single diagnostic peptide (i.e. 1453.7), which is common to several species (several carnivores, hare, and wild boar), the fragment was re-analysed morphologically. For 11 out of 13 samples, this approach allowed us to exclude the taxonomic attribution to *Lepus* sp., considering the size of the specimen and the thickness and compactness of the bone. For the samples that were too fragmented or did not provide any taxonomic indication, the category ‘Carnivora/*Sus scrofa*/*Lepus* sp.’ was retained. Only one sample was determined as *Bos/Bison* and 5 as *Sus scrofa*, which is consistent with data obtained from the zooarchaeological analyses. 9 bone fragments were identified as *Ursus* sp., which is widely represented in levels 1f and 1g of Riparo del Broion. *Ursus* sp. shares most of its diagnostic peptides with *Felis* sp. and *Lynx lynx*, and when the taxonomic assignment could not be improved molecularly, bone fragments were re-analysed morphologically to exclude unrelated species and improve the determination. *Felis silvestris* was often excluded due to its size and the thickness of the cortical bone, which were not consistent with the analysed fragments, thus 2 specimens were determined as *Ursus* sp./*Lynx lynx*. Finally, a specimen with anthropogenic slicing marks (Ril.989) was determined as a cervid, confirming what had previously been highlighted by the zooarchaeological analyses, namely the hunting and consuming of these animals by local hunter-gatherers.

#### Glutamine deamidation rate

All the analysed contexts display a glutamine deamidation rate (%Gln) between 0.27 and 0.38 for peptide with m/z 1105.6 and between 0.21 and 0.33 for peptide with m/z 1706.7 (where 0 indicates a fully deamidated sample and 1 when not deamidated at all). For both analysed peptides, the context with the highest mean deamidation is Uluzzo C (P1105.6: 0.29 ±0.02; P1706.7: 0.2 ±0.03), followed by Roccia San Sebastiano (P1105.6: 0.33 ±0.01; P1706.7: 0.21 ±0.01) and then by Riparo del Broion (P1105.6: 0.38±0.01; P1706.7: 0.33±0.01). We acknowledge that the different extraction methods employed (see Methods) might have influenced the deamidation level of collagen [49,50] since has been shown that HCl-treated samples returned greater levels of deamidation and a decrease in the number of peptides detected in the spectra [51].

## Discussion

### Combining ZooMS and morphological analyses: Faunal composition and variability

**Fig 2:**
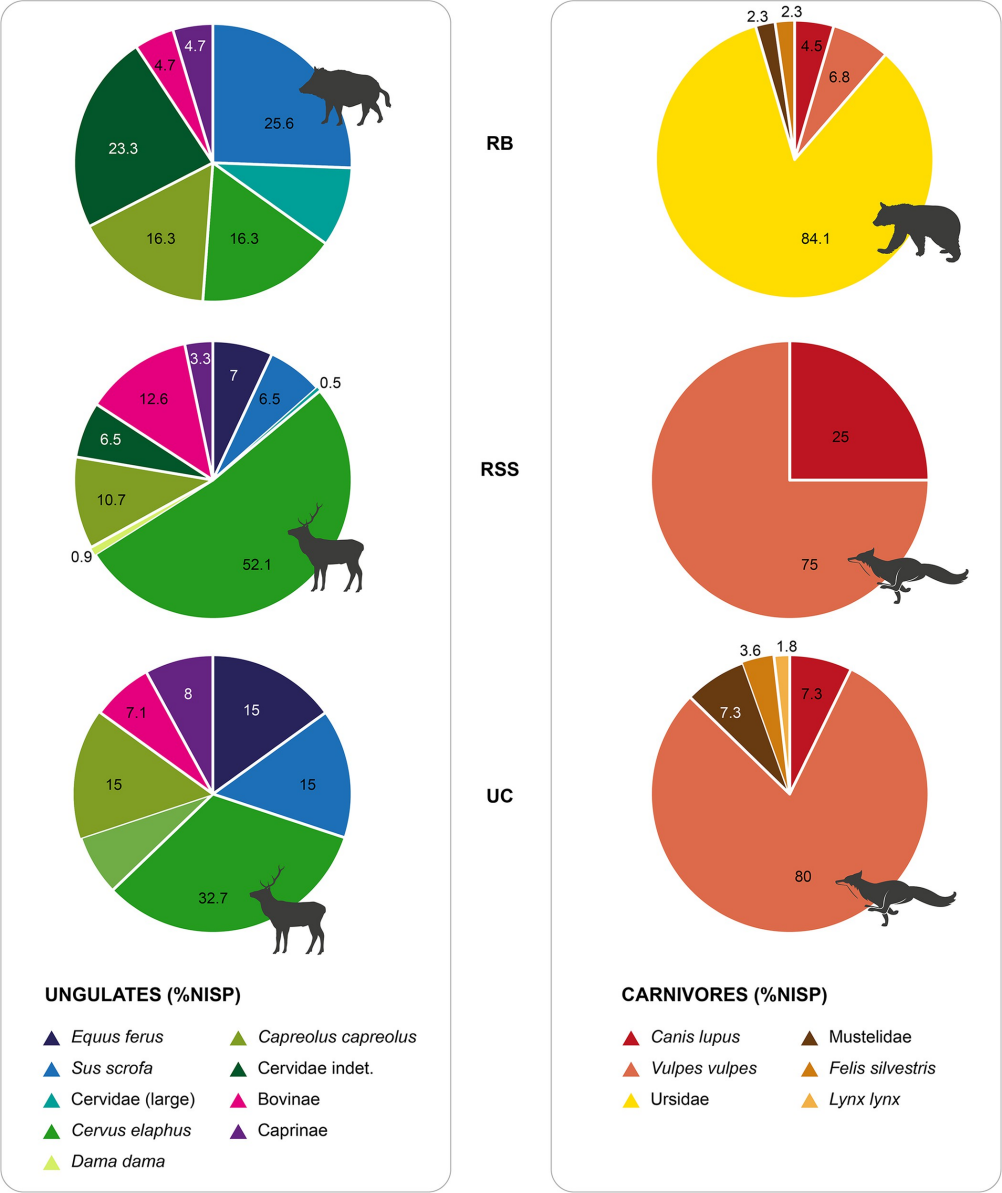
%NISP of Ungulates and Carnivores obtained from the zooarchaeological analysis. Percentages of Ungulates (%NISP) and Carnivores obtained from the morphological determination of the faunal assemblage for RB (Riparo del Broion), RSS (Roccia San Sebastiano cave) and UC (Uluzzo C Rock Shelter).

**Fig 3:**
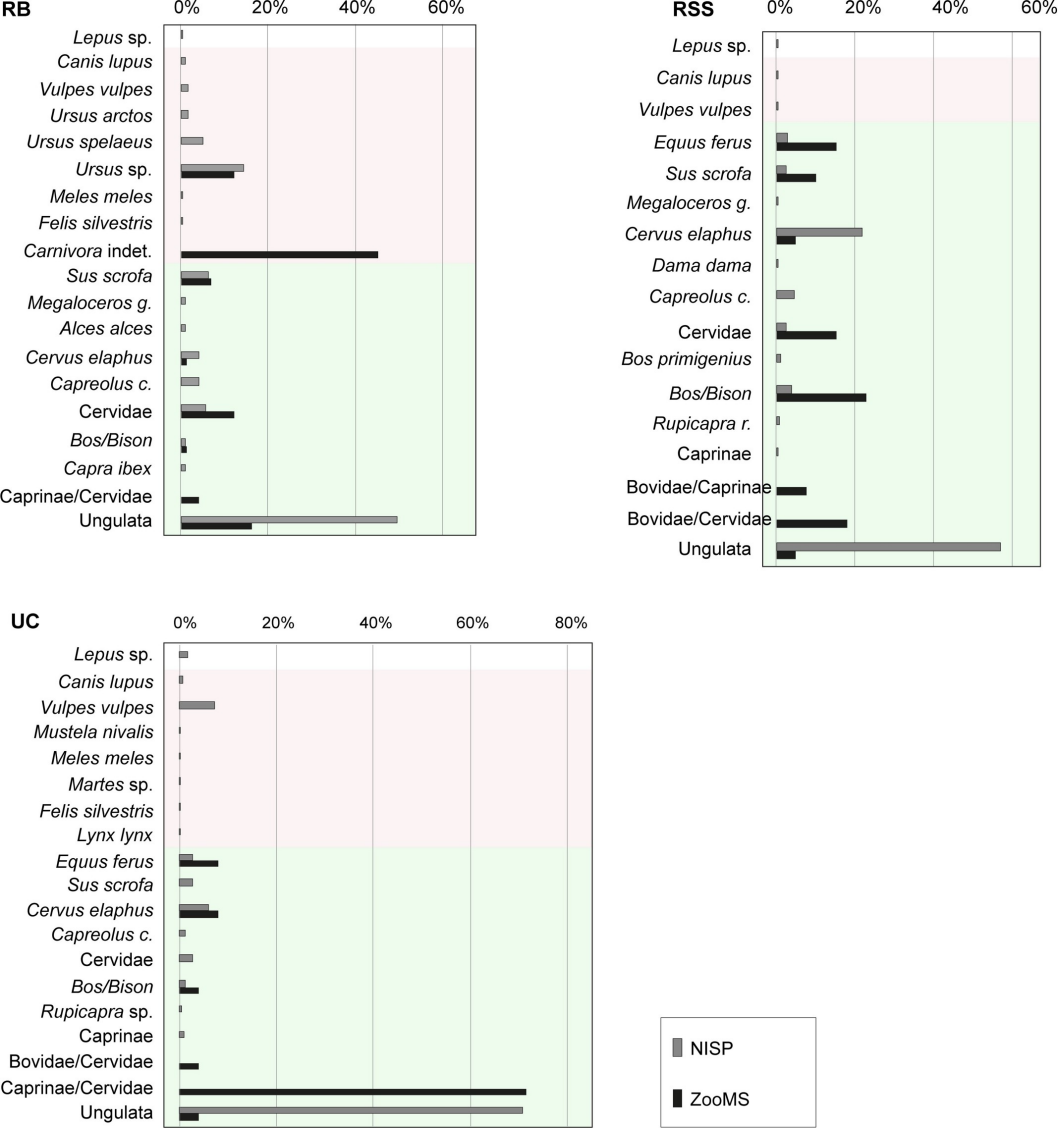
Taxonomic results from zooarchaeological identification and ZooMS. Comparison between the taxonomic determinations obtained through zooarchaeological analysis and ZooMS for the sites analysed (RB, UC and RSS).

Despite adopting the selection method designed for this study (see Methods), no human remains were identified. During the selection, we favoured bones with greater porosity and a more extensive presence of trabeculae on the cortical surface (morphological traits that are more characteristic to *Homo*) [57–59]. We noticed that the protocol followed for the selection of the samples involved a moderate sampling bias potentially limiting further zooarchaeological inferences. This is particularly evident with Riparo del Broion where–by focusing on specific characteristics of the bone fragments—we selected for the proteomic analysis mostly bones that have turned out to belong to carnivores, more similar to human bones than ungulates. Differences in the faunal composition of the sites were highlighted by zooarchaeological analysis (Fig 2). In general, ZooMS results are consistent with the data obtained from the morphological analyses (Fig 3), allowing to increase the NISP without identifying new taxa. For Uluzzo C, ZooMS was not pivotal since only 21 samples provided taxonomic attribution. Nevertheless, such a lack of molecular data indicates bad preservation of the collagen in the samples. Considering the warm climate of the area (17.6° C is the recorded average annual temperature [60]) the proximity to the sea, the state of preservation of the remains (i.e. frequent and abundant carbonatic concretions), and the alkaline nature of the sediment with a high amount of carbonates [40], the scarcity of collagen was expected since these soil characteristics are known to induce the degradation of the organic matter in the samples [61–64]. At Roccia San Sebastiano the better organic preservation of the samples compared to Uluzzo C yielded more data from both the morphological analysis and the ZooMS. The faunal assemblages of Uluzzo C and Roccia San Sebastiano cave are very similar (Fig 2) since both are located in southern Italy. As far as Riparo del Broion is concerned, ZooMS results highlight the presence of several samples of bear, which were also abundant in the morphological analysis, 5 remains of wild boar and 9 of indeterminate cervids. Then, a single fragment of bovid was identified, which is also consistent with the zooarchaeological results. ZooMS analysis increases the number of indeterminate carnivores (Fig 3) and the general NISP (from 638 fragments being taxonomically determined with the morphological method, to 750). No additional taxa were identified, similarly to previous preliminary ZooMS analysis conducted on a sample of 743 bones [65].

**Fig 4:**
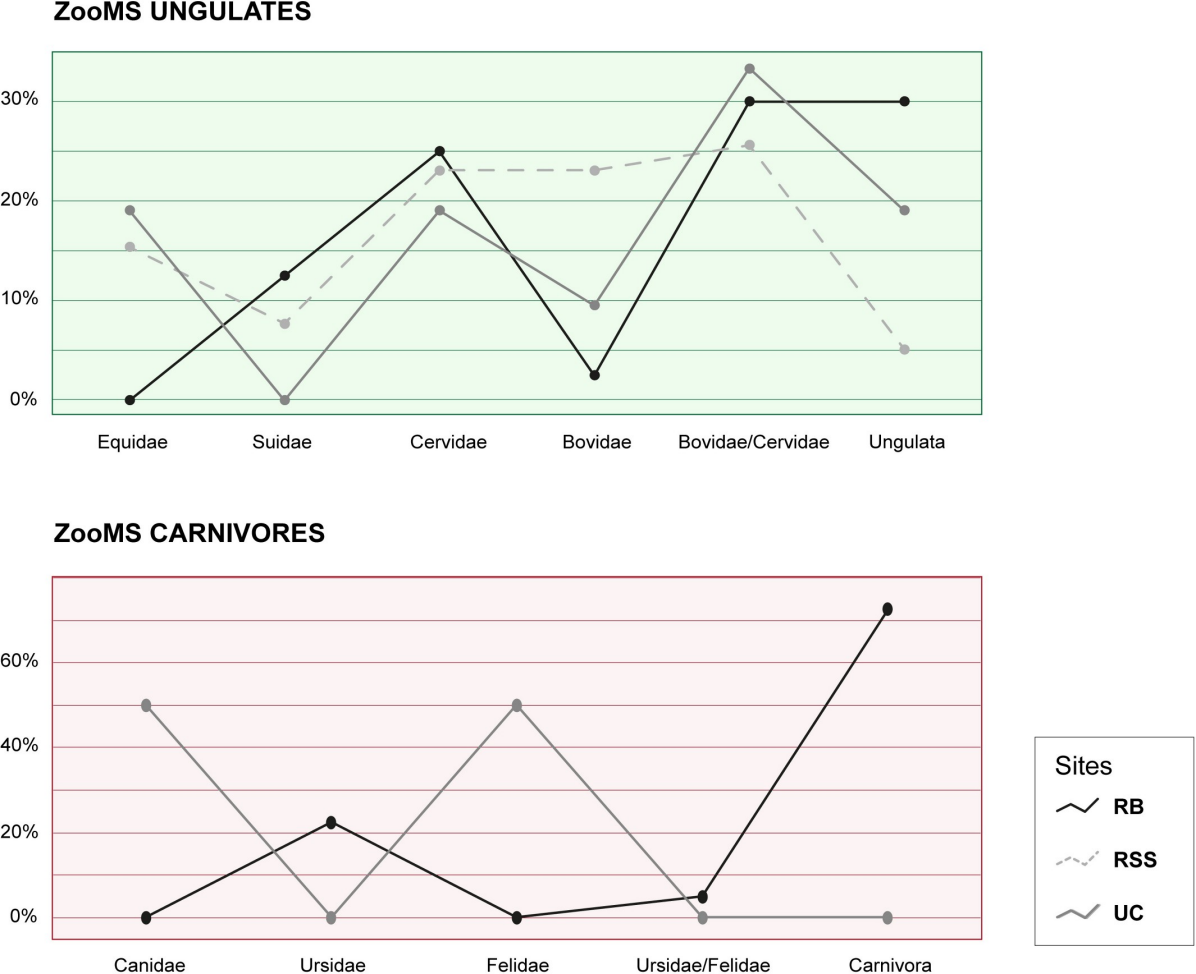
Percentages of Ungulates and Carnivores obtained by ZooMS for RB (Riparo del Broion), RSS (Roccia San Sebastiano cave) and UC (Uluzzo C Rock Shelter).

The sites show a remarkable difference in terms of faunal composition, and this is evident from the morphological analysis as well as from the ZooMS analysis, whose results are coherent with what emerges from the archaeozoological analysis of the specimens. As mentioned above, at Roccia San Sebastiano cave, few bone fragments of small-sized species were found, and only 2 bone fragments of hare were recorded. Among the carnivores, we identified a single bone fragment of wolf. By contrast, at Uluzzo C a significant presence of hares, mustelids and carnivores of small-medium size was recorded (Fig 3). Ungulates are represented in both sites, in particular red deer. At Roccia San Sebastiano cave we also registered fallow deer and large-sized cervids such as giant deer. Less abundant in both sites are caprines, whereas on the other hand a huge quantity of cattle and wild boar remains were registered. In both sites, ungulates are more abundant than carnivores (Fig 4).

### The Uluzzian framework in Italy

The Uluzzian framework of our three assemblages includes a few sites. In Southern Italy, Grotta del Cavallo (Lecce) showed a paucity of *Lepus* sp. and carnivores, akin to the investigated contexts. This site preserved a higher number of horses, red deer, and bovids remains, but a lower number of wild boars compared to Uluzzo C and Roccia San Sebastiano cave [26]. Similarly, Grotta di Castelcivita (Salerno) is characterised by a remarkable number of horses and large bovid remains [25,66]. At Grotta della Cala (Marina di Camerota, Salerno) a conspicuous presence of cervids, and especially of the fallow deer, has been reported (akin to Roccia San Sebastiano cave) [67]. In contrast, in Northern Italy, Riparo del Broion is characterised by a different faunal composition compared to the previously mentioned sites. The amount of carnivore remains is conspicuous, especially *Ursus* sp. (*arctos* and *spelaeus*), whereas mustelids are less abundant compared to Uluzzo C. Likewise, the total amount of hare and fox remains is scarce. Among ungulates, which are, however, the largest group represented, we found cervids of different sizes: elk (absent in the other two southern contexts), giant deer (also present at Roccia San Sebastiano cave), red deer and roe deer. The wild boar is the second most abundant species, after cervids. The number of recorded bovids decreases considerably at Riparo del Broion. This context is the sole recording the presence of goats, due to rocky areas surrounding the site. Moreover, the faunal composition from Fumane cave is similar and is characterised by large carnivores such as ursids, and small/medium-sized carnivores such as foxes and wolves; cervids of various sizes, bovids and ibex are the most common ungulate species; and mustelids or small species are not attested [7,44,68–71].

The taxonomic spectra of the sites, from both the north and the south, show a remarkable diversity of carnivore and ungulate species, except for the scarce presence of carnivore taxa at Roccia San Sebastiano cave. Indeed, the presence of ungulate remains, as well as some carnivores, is linked to human subsistence activities, who exploited different biotopes in proximity of the sites [25]. On the other hand, the presence of the above-mentioned species could provide a well-established paleoenvironmental proxy for the Uluzzian period based on the ecological requirements of the different species. Altogether our data indicate that across the Uluzzian transition, the climate in Italy was mainly cold-temperate (Table 3). For Roccia San Sebastiano cave, the presence of horses, red deer, wild boar, and bovids indicate wooded meadows and open spaces. The presence of *Rupicapra* sp. could be correlated to an increase in humidity. The high frequency of horses at Uluzzo C suggests the occurrence of sparse woodland and steppic environments, as well as the presence of Cervidae, which are typical of Mediterranean evergreen forests [24]. At Riparo del Broion, the conspicuous presence of *Ursus* sp. indicates humid conditions and open environments. Humid woodlands are suggested also by the presence of moose, giant deer and wild boar. The presence of ibex is indicative of an alpine setting. In summary, mammal assemblages show that the Middle-Upper Palaeolithic transition was associated with a shift to colder climatic conditions, according to [25].

**Table 3:** Sites under analysis with reference to the dominant taxa and to the most represented environmental and climate setting.

Localization	Sites	Taxa	Climate/Environment
Northern Italy	Riparo del Broion	*Ursus* sp.	cold-temperate climate with humid conditions and open environments
		*Alces Alces*	temperate, boreal forest, and tundra environments with humid woodlands and areas near water
		*Megaloceros giganteus*	cold climate with mixed forest-tundra environments
		*Sus scrofa*	cold-temperate climate with humid woodlands
		*Capra ibex*	cold climate with steppic environments
Southern Italy	Roccia San Sebastiano cave	*Equus ferus*	cold climate with presence of open spaces
		*Cervus elaphus*	temperate climate with wooded meadows and open spaces
		*Sus scrofa*	cold-temperate climate with humid woodlands
		*Bos/Bison*	cold-temperate climate with wooded meadows and open spaces
		*Rupicapra* sp.	temperate climate with an increase in humidity, alpine and forest areas
	Uluzzo C Rock Shelter	*Equus ferus*	sparse woodland and steppic environments
		*Cervus elaphus*	temperate climate with wooded meadows and open spaces
		*Capreolus capreolus*	cold-temperate climate with open woodland areas

### Glutamine deamidation and context variation

**Fig 5:**
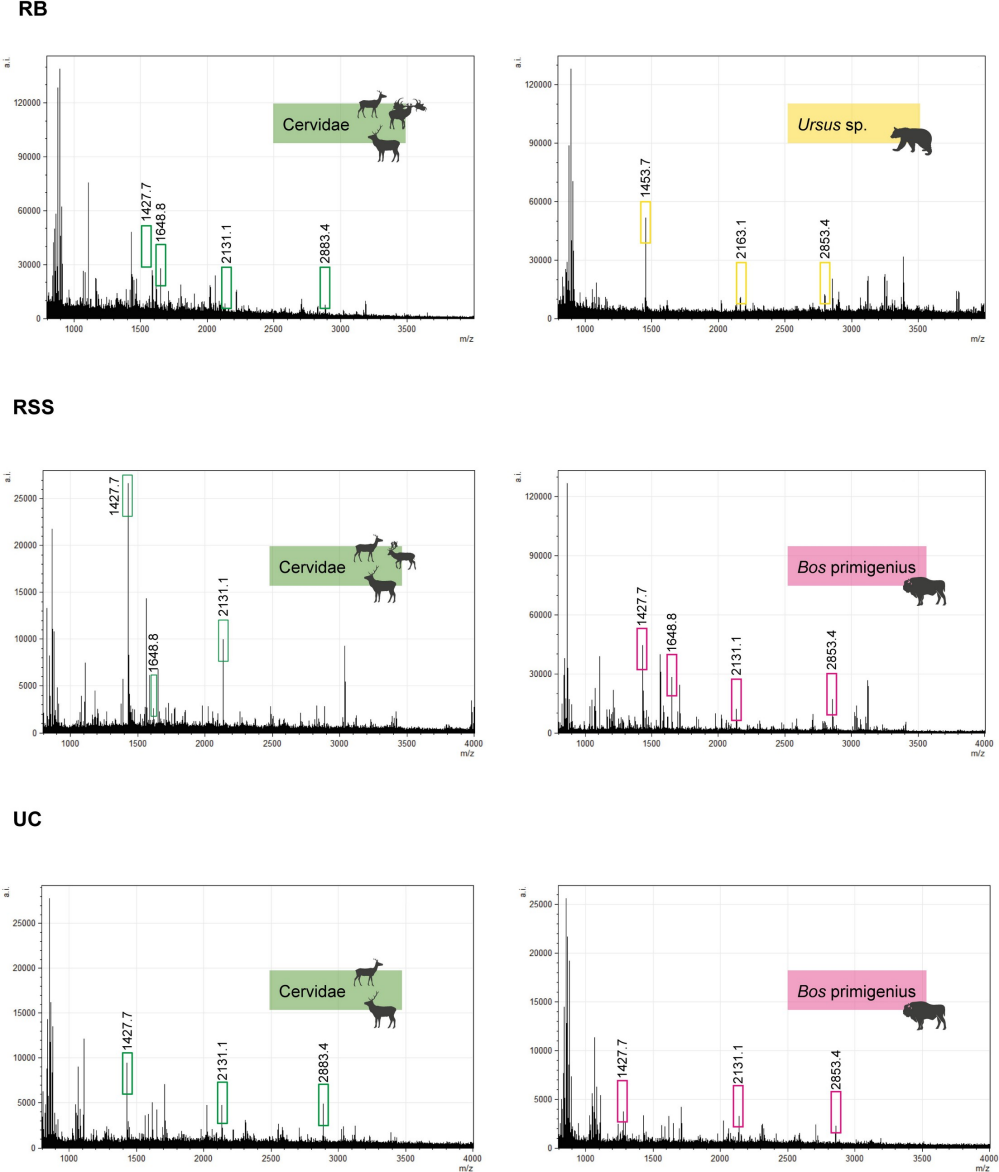
MS spectra from the different sites. RB (Riparo del Broion), RSS (Roccia San Sebastiano cave) and UC (Uluzzo C Rock Shelter). Peptide markers for the taxonomic determination have been highlighted in the spectra.

The highest quality spectra are from Riparo del Broion and Roccia San Sebastiano cave, despite the latter being located in southern Italy. Conversely, a different situation in terms of quality and legibility of the spectra (e.g., high baseline, ground noise, several undiagnostic peptides, averagely lower peaks, and few diagnostic peptides for the taxonomic determination) is outlined for Uluzzo C (Fig 5) where the samples were particularly dehydrated and concreted, with an evident compromised taphonomic state. It has been demonstrated that the loss of collagen in a bone sample is linked to various factors such as time, temperature, humidity of the burial environment and pH of sediment. Extreme temperatures and pHs can accelerate protein hydrolysis [72]. In addition, the microbial decomposition of bone can be strongly influenced by factors affecting the burial environment such as oxygen or the presence of water [73]. This could explain the lack of collagen and the high percentage of indeterminate fragments (higher than 50%) in a context such as Uluzzo C, a site located in southern Italy in proximity of the sea, unlike Roccia San Sebastiano cave. However, it should also be noted that not all the samples considered in the calculation were subjected to the same extraction protocol.

**Fig 6:**
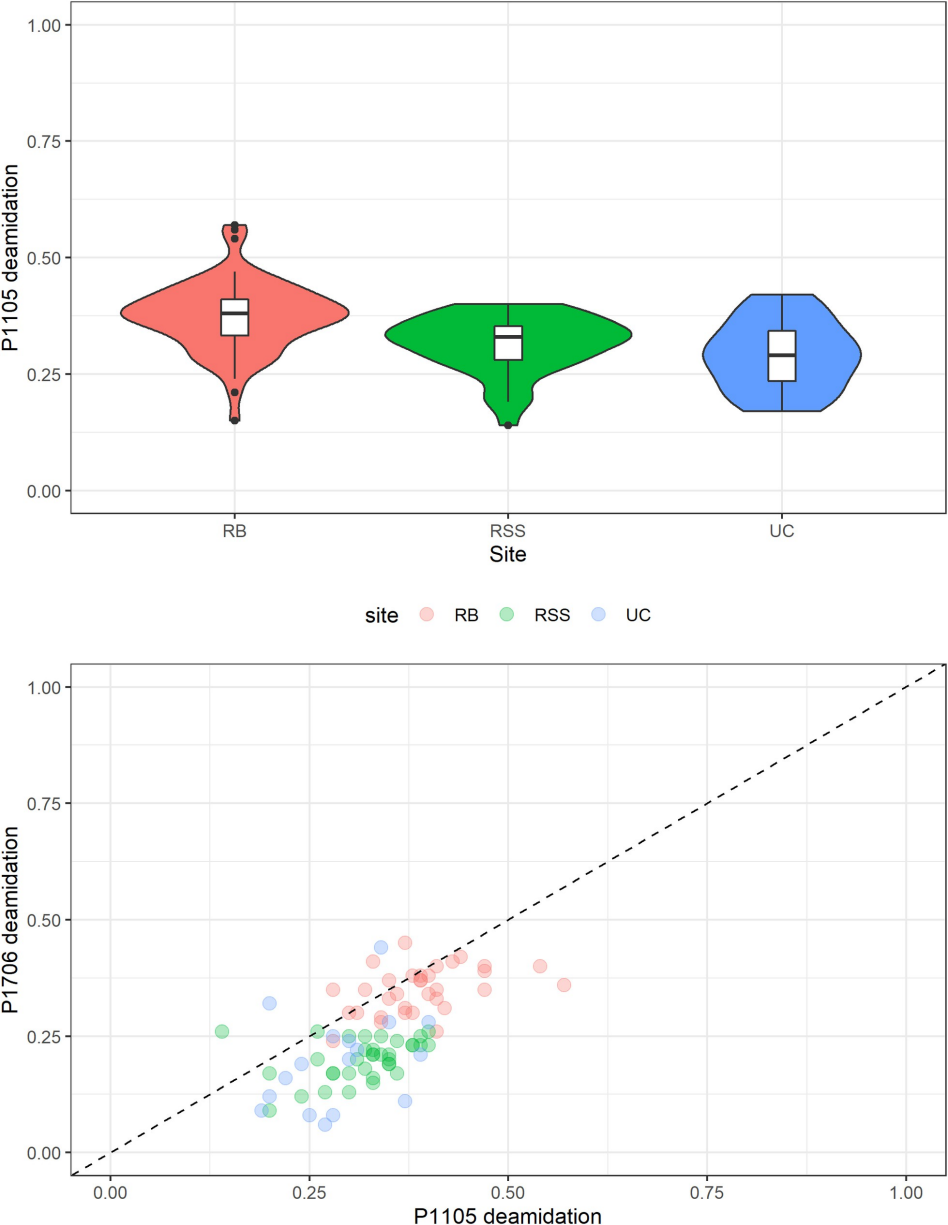
Violin plot and biplot of glutamine deamidation ratios. %Gln of P1105 for RB (Riparo del Broion), RSS (Roccia San Sebastiano cave) and UC (Uluzzo C Rock Shelter) and biplot of %Gln of P1105 and %Gln of P1706 for all the analysed contexts, the dashed line is the 1:1 ratio.

Glutamine deamidation ratios obtained for the contexts considered in this study range between 0.29 and 0.38 for peptide with m/z 1105.6 (mean: 0.33) and between 0.20 and 0.32 for peptide 1706.7 (mean: 0.26), indicating that the samples are heavily deamidated (Fig 6). This is particularly evident when data are compared with values from Denisova cave (%Gln between 0.4 and 0.8) [74], the Châtelperronian layers of Quinçay, France (%Gln P1105 between 0.1 and 0.9, mean 0.5) [36] and the Châtelperronian layers of Grotte du Renne, France (mean %Gln P1105: 0.15; P1706: 0.45) [9]. In addition, our values are coherent with %Gln obtained for the Uluzzian layers of Fumane cave, northern Italy (between 0.2 and 0.7) [7].

In this study, we found differences in the quantity and quality of taxonomic data among the different contexts (Fig 5). The scarcity of taxonomic results obtained for Uluzzo C compared to Riparo del Broion, is reflected in the percentage of deamidation which was obtained. Data obtained from the calculation of deamidation are coherent with the results of taxonomic determinations. By comparing the deamidation percentage in the different contexts with the determination success rate in the determinations obtained (Table 4), it can be noted that Uluzzo C (with high deamidation on average P1105.6: 0.29 ±0.03 and P1706.7: 0.20 ±0.05) returned a very low success rate in the taxonomic determination by ZooMS. On the other hand, the mean of deamidation at Riparo del Broion is the lowest among the analysed contexts and the taxonomic determination percentage of success is the highest recorded. The variation in %Gln might reflect localized geological and environmental conditions, which differ among the sites taken into consideration in this study. Since deamidation is temperature and pH dependent [75–78], we suggest that the favourable environmental condition of Riparo del Broion ensured collagen preservation in the analysed bones, ultimately allowing us to obtain further taxonomic information from this site.

**Table 4:** Deamidation medians and means for the two peptides taken into consideration (peptides with m/z 1105.6 and P1706.7) with the respective 2-standard error (reported as 2 SE) and the ZooMS taxonomic determination success rate in the taxonomic determination for all the analysed contexts.

	P1105.6			P1706.7			
Site	median	mean	2 SE	median	mean	2 SE	ZooMS success rate
Riparo del Broion	0.38	0.38	0.02	0.34	0.32	0.02	90.8
Roccia San Sebastiano	0.33	0.32	0.02	0.21	0.20	0.01	74.5
Uluzzo C	0.29	0.29	0.03	0.20	0.20	0.05	36.9

## Conclusions

In this study, the combination of traditional zooarchaeology and proteomic analyses allowed us to give a more exhaustive interpretation to the various contexts and to propose a new method of sample selection for ZooMS analyses which focuses only on samples that correspond to specific morphological characteristics. Here we avoided the massive bone sampling adopted in other works (e.g. Fumane cave; [7]) and favoured a preliminary accurate morphological observation and selection of the samples. This method avoided the destructive sampling of all the unidentifiable fragments, focusing only on the ones that met specific characteristics for the obtainment of the set results, among which was identifying potential human specimens. The two approaches employed (traditional and proteomic) supported each other. When the ZooMS identification remained quite generic, the re-analysis of the fragment from a morphological point of view allowed us to exclude certain taxa and to obtain a more specific determination. In cases of ambiguity in the taxonomical identification through morphological analysis (e.g., undiagnostic diaphysis of mammals of the same size), ZooMS was useful to clarify the taxon of the sample. As mentioned above, however, the protocol followed for the selection of the samples likely involved a moderate sampling bias, potentially limiting additional zooarchaeological inferences (such is particularly evident with Riparo del Broion). This is an important consideration to bear in mind if the study aims to reconstruct (without the support of zooarchaeological analysis) the faunal composition of the site investigated, but the approach can be useful if the goal is to identify human specimens, since it excludes many ungulate bone fragments. This method allowed us to increase the NISP in all analysed contexts, in one considerably (Riparo del Broion), whereas in the others relatively less due to the poor preservation of the collagen. The application of ZooMS in such contexts provided new data, expanding our understanding of the faunal record and of the paleoenvironmental and paleoclimatic conditions. The different success rates are illustrative of the difference in diagenetic alterations among the analysed sites, providing information on each context’s suitability for further analyses (DNA, radiocarbon dating). Further zooarchaeological and taphonomic analyses are ongoing, aiming to implement our knowledge on the subsistence activities and occupations patterns adopted by human groups during the Middle-Upper Palaeolithic in Italy.
